# Limited genomic signatures of population collapse in the critically endangered black abalone (*Haliotis cracherodii*)

**DOI:** 10.1111/mec.17362

**Published:** 2024-04-29

**Authors:** Brock Wooldridge, Chloé Orland, Erik Enbody, Merly Escalona, Cade Mirchandani, Russell Corbett-Detig, Joshua D. Kapp, Nathaniel Fletcher, Karah Cox-Ammann, Peter Raimondi, Beth Shapiro

**Affiliations:** 1. Ecology and Evolutionary Biology Department, University of California Santa Cruz, Santa Cruz, CA, 95064 USA; 2. Howard Hughes Medical Institute, University of California Santa Cruz, Santa Cruz, CA, 95064, USA; 3. Department of Biomolecular Engineering, University of California Santa Cruz, Santa Cruz, CA, 95064 USA; 4. Genomics Institute, University of California Santa Cruz, Santa Cruz, CA, 95064 USA

**Keywords:** conservation genetics, population bottleneck, black abalone, chromosomal inversion

## Abstract

The black abalone, *Haliotis cracherodii*, is a large, long-lived marine mollusc that inhabits rocky intertidal habitats along the coast of California and Mexico. In 1985, populations were impacted by a bacterial disease known as withering syndrome (WS) that wiped out >90% of individuals, leading to the closure of all U.S. black abalone fisheries since 1993. Current conservation strategies include restoring diminished populations by translocating healthy individuals. However, population collapse on this scale may have dramatically lowered genetic diversity and strengthened geographic differentiation, making translocation-based recovery contentious. Additionally, the current prevalence of WS is unknown. To address these uncertainties, we sequenced and analyzed the genomes of 133 black abalone individuals from across their present range. We observed no spatial genetic structure among black abalone, with the exception of a single chromosomal inversion that increases in frequency with latitude. Outside of the inversion, genetic differentiation between sites is minimal and does not scale with either geographic distance or environmental dissimilarity. Genetic diversity appears uniformly high across the range. Demographic inference does indicate a severe population bottleneck beginning just 15 generations in the past, but this decline is short-lived, with present day size far exceeding the pre-bottleneck status quo. Finally, we find the bacterial agent of WS is equally present across the sampled range, but only in 10% of individuals. The lack of population genetic structure, uniform diversity, and prevalence of WS bacteria indicates that translocation could be a valid and low-risk means of population restoration for black abalone species’ recovery.

## 1 Introduction

Severe population declines threaten a species’ long-term viability and can even result in extinction. Although conservation of remnant populations is essential to maintain any hope of recovery, a key question is whether the genetic effects of such a decline (i.e. bottleneck) are too deleterious to overcome (Robinson et al. 2022). Smaller populations are expected to experience inbreeding depression, or reduced fitness as a result of increased mating between related individuals (D. Charlesworth and Willis 2009; Keller and Waller 2002). Natural selection is thought to be less effective at removing mildly deleterious alleles in small populations, permitting the accumulation of deleterious variation (Agrawal and Whitlock 2012; Lynch, Conery, and Burger 1995). Finally, reduced overall variation in smaller populations is also thought to limit the potential to adapt to new environments (B. Charlesworth 2009; Frankham et al. 1999; Hoffmann, Sgrò, and Kristensen 2017). While the relationship between these phenomena, i.e. genetic diversity and extinction risk, is complex (Kardos et al. 2021; Teixeira and Huber 2021), the field of conservation genomics continues to play an essential role in guiding the recovery of small populations (Shaffer et al. 2022).

Genomic data can provide surprising insights into the status of small populations. Recent work in the vaquita porpoise, of which there are approximately 20 individuals left in the wild, revealed unexpected large historical effective population size (N_e_ > 1000) and a limited effect of recent decline on the accumulation of deleterious alleles, thereby demonstrating an unexpected potential for recovery (Robinson et al. 2022). Similarly, genomic data have shown that long-term small population size has actually enabled Channel Islands foxes to effectively purge highly deleterious variants, suggesting that genetic rescue through introduction of new individuals may not be an appropriate strategy (Robinson et al. 2018). Even when expected, the outcomes of conservation genomics studies can still provide valuable guidance for species management, either through quantifying mutation load and identifying deleterious alleles (Tian et al. 2022) or delineating thresholds for minimum population size to avoid further inbreeding depression (Grossen et al. 2020). However, these genomic metrics of population health may not directly translate to viability, as evidence of organismal fitness is often lacking (but see (Yates, Bowles, and Fraser 2019). Still, genomic data can provide a helpful lens to understanding the trajectory of a threatened population (Willi et al. 2022). With genomic resources becoming increasingly available for non-model organisms, including those that were once prohibitively difficult to access and sequence, it becomes imperative to integrate this information into management and recovery strategies.

Black abalone (*Haliotis cracherodii*, Leach 1814) are large, long-lived marine gastropods found along roughly 1,500 km of the coastline from Point Arena, in California, USA, to Bahia Tortugas and Isla Guadalupe, in Baja California, Mexico (Neuman, Tissot, and VanBlaricom 2010). They typically live in rocky intertidal habitats and less often subtidally to a depth of six meters, and occupy a key niche in intertidal ecosystems as primary consumers of macroalgae (Leighton and Boolootian 1963) and common prey items for sea otters (Raimondi, Jurgens, and Tinker 2015). Black abalone facilitate encrusting coralline algae, thereby maintaining favorable habitat for conspecific recruitment on rocky intertidal reefs (Miner et al. 2006; Cox 1962; Richards and Davis 1993). Black abalone are dioecious and reproduce by broadcast spawning; while this reproductive strategy may facilitate gene flow between distant populations, the negative buoyancy of embryos and the 5–15 day larval swimming phase that follows is thought to limit dispersal in comparison to other broadcast spawners (Chambers et al. 2006).

In addition to playing a key ecosystem role, the abundance, accessibility, and size of black abalone have made them a frequent target of human populations for at least 13,000 years (Haas et al. 2019). The meaty foot has served as a food staple for indigenous Californians, and their iridescent shells have been used as adornments, tools, cultural currency, and religious symbols (Erlandson et al. 2008; Kelley and Francis 2003; Sloan 2003; Vileisis 2020). After colonists from Spain and the United States replaced indigenous peoples through displacement, disease, and violence from the 17th-19th centuries, abalone were thereafter heavily impacted by commercial fishing (Bentz and Braje 2017; Braje et al. 2014). These fisheries produced catches that are inconceivable today; in 1973 alone around 800 tons of black abalone were harvested from the California Channel Islands (Karpov et al. 2000).

Despite black abalone being arguably the hardiest of the seven abalone species found along this coastline (Tissot 1988; Vileisis 2020), black abalone were nearly erased during the late 20^th^ century (Rogers-Bennett 2002). While intensified fishing and environmental pressures (e.g. oil spills, sea temperature rise, sediment burials) contributed to this decline, the primary culprit was the emergence of a devastating disease known as withering syndrome (WS). WS is caused by the Rickettsia-like bacteria *Candidatus Xenohaliotis californiensis*, also known as WS-RLO, which attacks the lining of the digestive tract, resulting in reduced body mass and eventual withering of the abalone’s foot until it can no longer cling to the substrate (Lafferty and Kuris 1993; C. S. Friedman et al. 2000). Following the onset of WS around 1985, black abalone underwent widespread mass mortality events. In areas most affected by the disease, populations declined by up to 99% (Neuman, Tissot, and VanBlaricom 2010; VanBlaricom et al. 2009; Crosson et al. 2014). These dramatic declines led to the closure of all black abalone fisheries in 1993, and in 2009 the species was listed as endangered under the U.S. Endangered Species Act. Changes to intertidal ecosystems across the range followed this collapse, with habitats previously dominated by crustose coralline algae and bare rock becoming overgrown with fleshy algae and sessile invertebrates (Miner et al. 2006).

WS has been most severe in populations south of California’s Point Conception, including the California Channel Islands, and appears to be exacerbated in populations experiencing anomalously warm water (Ben-Horin, Lenihan, and Lafferty 2013; Crosson and Friedman 2018), following warm El Niño cycles (Raimondi et al. 2002) or from power plant outflows (Altstatt et al. 1996). However, the mechanisms governing susceptibility are largely unresolved, in large part due to the overwhelming severity of the disease and the lack of suitable populations for study. The recent recovery of some populations indicates that WS immunity may exist, and this immunity has been tenuously linked to both a) a phage that infects the WS-causing bacteria and b) heritable genetic variation. Observations of a phage hyperparasite, known as *Xenohaliotis* or RLOv, infecting the bacteria led to the discovery that phage presence appears to partially attenuate WS development, but like the primary abalone-Rickettsia relationship this effect is also dependent on temperature (Carolyn S. Friedman et al. 2014). At the same time, some populations - and other species - appear to have evolved limited resistance to WS even in the absence of phage (Brokordt et al. 2017; Crosson et al. 2014). However, evidence for both phenomena is preliminary, and it is unclear to what extent black abalone across the range have adapted to the spread of WS.

**Figure 1.**
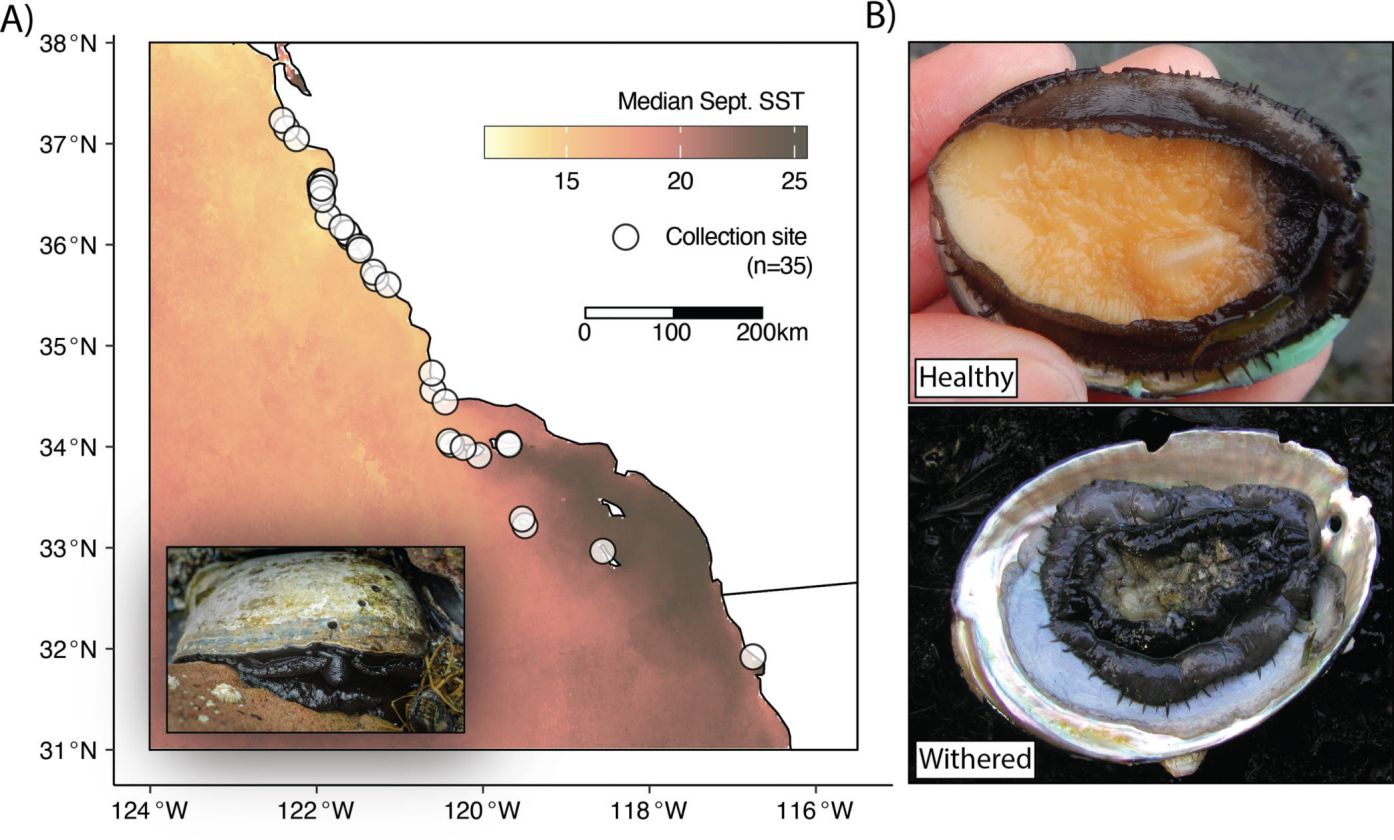
**A)** Distribution of collection sites along the California and Baja California coast. Inset displays adult black abalone, image by Michael Ready ©. **B)** Representative images of healthy and withered black abalone. Photos by Nathaniel Fletcher.

It is against this backdrop of overfishing, disease spread, and rapid decline that a genetic approach to species conservation has become particularly needed. Although substantial effort has gone towards understanding the status of remaining populations, less has been done through the lens of conservation genetics. Encouragingly, previous studies in black abalone have reported limited population genetic structure and isolation-by-distance in remnant populations, although these studies are constricted in both geographic range and the molecular markers assayed (Gruenthal and Burton 2008; Hamm and Burton 2000; Chambers et al. 2006). Other abalone species, including the subtidal green abalone (Gruenthal et al. 2014) and red abalone (Gruenthal, Acheson, and Burton 2007) have also shown a lack of differentiation and isolation-by-distance in their California ranges. Despite this agreement, biotic factors like fecundity, planktonic duration, and preferred habitat indeed vary between California abalone, and can therefore be expected to influence genetic structure (Kelly and Palumbi 2010; Dawson 2001; Vileisis 2020). Abiotic factors are also liable to influence genetic structure on the California coast. While the California Current helps to distribute propagules along the entirety of the coastline, this flow is generally stronger from the north to south and varies by season, which could contribute to asymmetry in connectivity between sites. Additionally, prominent geographic features like Point Conception represent major regime changes in environmental conditions (e.g. sea temperature; Fig. 1A) and may provide significant barriers to gene flow at particular times of the year (Dawson 2001; Hohenlohe 2004).

Whole-genome data from wild black abalone could help address these uncertainties by quantifying remaining genetic diversity, connectivity between sites, and genetic associations with population success. This information combined with decades of ecological research will help guide critical management decisions, including planned translocations of which there have been almost none to date (Orozco 2023). To this end, we developed a low impact external swabbing method for obtaining whole-genome data from wild black abalone, and sequenced 133 individuals from across ~800 km of the former range. With these data we present the most comprehensive picture yet of population structure in this critically endangered species. Additionally, we explore how patterns of gene flow and genetic diversity associate with geographic and environmental variation to assess the extent to which the few remaining populations are genetically isolated. Finally, we determine the distribution of the bacterial agent of withering syndrome and explore associations with its phage hyperparasite.

## 2 Results

### 2.1 Whole genome sequencing of black abalone via DNA swabs

Because black abalone adhere strongly to their substrate, injuries during removal are often fatal. Therefore, obtaining substantial tissue clips can risk serious harm to the animal. To avoid this risk, we tested whether high-quality whole genomes could be obtained from swabbing the exposed edge of an individual’s foot with a sterile nylon-tipped swab. Using this approach, we swabbed 150 healthy abalone across 35 sites spanning the black abalone range (Fig. 1) and obtained high-quality DNA extracts from 133. The average DNA content of sequencing libraries prepared from these swabs was 63% black abalone, with significant proportions of reads mapping to each individual’s internal and external microbiome, including known parasites and symbionts (see section 2.5). Libraries were sufficiently complex to generate 5–20-fold genomic coverage per individual.

### 2.2 Population structure along the California coast

**Figure 2.**
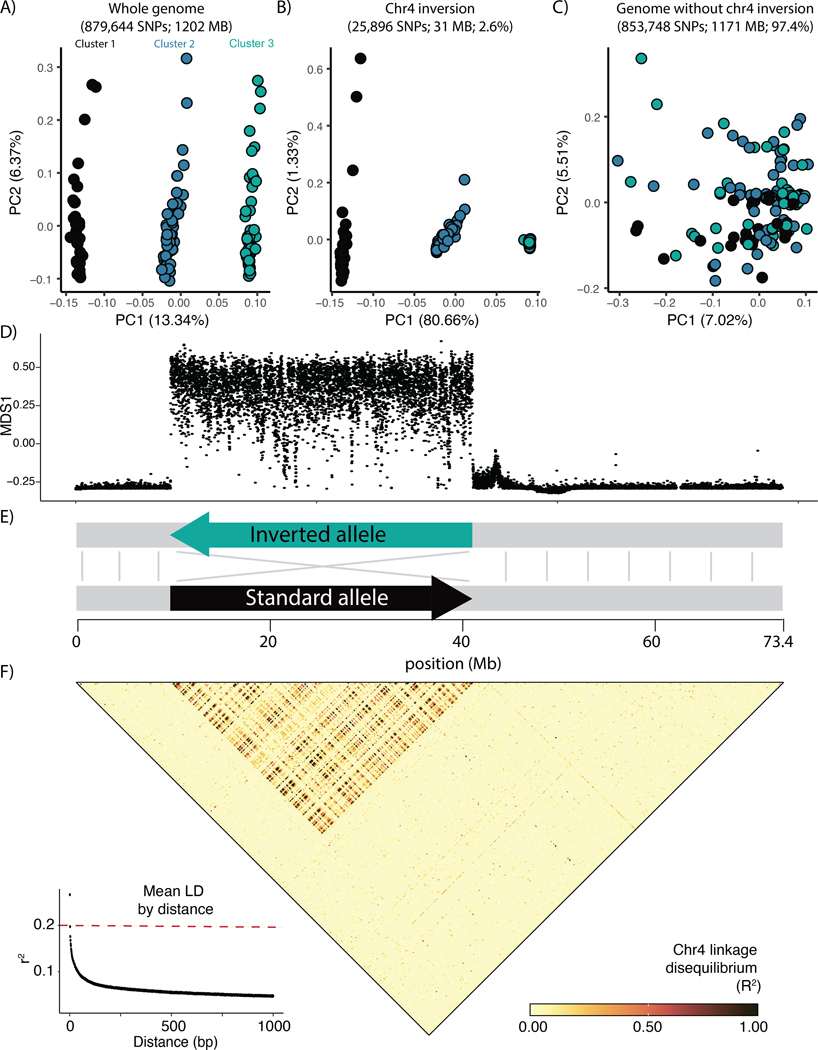
**A)** Genetic PCA based on genome-wide SNPs. In this panel colors are arbitrarily assigned to the three primary clusters., **B)** Genetic PCA based on SNPs from the putative chromosomal inversion only. Colors here correspond to those initially assigned in the genome-wide PCA, demonstrating the one-to-one mapping between cluster membership in panel A and inversion genotype in panel B. **C)** Genetic PCA based on genome-wide SNPs excluding the chr4 inversion. Colors here correspond to those initially assigned in the genome-wide PCA (2A). **D)** Sliding-window PCA (*lostruct*), each point corresponds to the local genomic structure in 5000 bp genomic windows, with the “MDS1” position roughly representing a snapshot of local population structure. **E)** Simplified schematic showing size and structure of chr4 inversion, with the colors of the inverted and reference alleles corresponding to the colors of inversion and reference homozygous individuals in panel B. **F)** Heatmap showing linkage disequilibrium across all samples for chr4. Lower left inset displays decay in linkage disequilibrium as a function of genomic distance in base pairs. Value represents mean r^2^ across chromosomes excluding chr4, and the red horizontal line at 0.2 indicates a common cutoff by which SNPs are typically considered uncorrelated (Hahn 2018).

A principal components analysis of genome-wide SNPs showed all samples clustering into one of three discrete groups along the first principal component (PC1), which explained 13.34% of the variation in the data (PC1; Fig. 2A). Within each cluster, samples also showed variation along PC2, although this principal component only accounted for 6.37% of the variation in the data. To understand what may be driving this unusual and highly structured pattern, we performed local sliding-window PCA with *lostruct* (Han Li and Ralph 2019) and identified a large 31MB region on chromosome 4 significantly impacting genome-wide population structure (Fig. 2D). Interestingly, a PCA of SNPs from this 31MB region (Fig. 2B) showed identical group membership to the whole-genome PCA (Fig. 2A), suggesting that population structure at this region alone is driving the whole-genome pattern along PC1. When we excluded this region - which comprises 2.6% of the genome - from our analysis, the signal of clustering along PC1 disappeared, resulting in a cloud of samples with minimal discernible structure (Fig. 2C).

Further analysis of this anomalous region revealed a large chromosomal inversion (Fig. 2E). A Hi-C contact map based on the diploid reference genome (Orland et al. 2022) showed two potential scaffold arrangements directly corresponding to the distinct *lostruct* peak at chr4: 9.8 – 41.2 Mb (Fig. S1A). These two alternative arrangements map in opposite directions to each other, suggesting that this reference genome is heterozygous for a chromosomal inversion. Long-read variants also point towards an inversion in this region, although the suggested breakpoints only roughly correspond to those hinted at by the prior analyses (Fig. S1B). Finally, linkage-disequilibrium (LD) analysis of all 133 sequenced individuals showed LD within the putative inversion boundaries to be elevated far above the genome-wide baseline, as might be expected if recombination-suppressing inversions were present (Fig. 2F; (Hager et al. 2022). Together, these lines of evidence suggest that a 31MB non-recombining inversion on chr4 is underlying the three distinct clusters in the whole-genome PCA. This inversion is polymorphic in our samples (allele frequency = 58%); while the ancestral state is not known, the severely reduced polymorphism in the right homozygous group (Fig. 2B; Fig. S3) is suggestive of a recent origin and possibly selection, leading us to designate that allele as “inverted” and the alternate allele as “standard” (Fig. 2E; (Guerrero, Rousset, and Kirkpatrick 2012).

**Figure 3.**
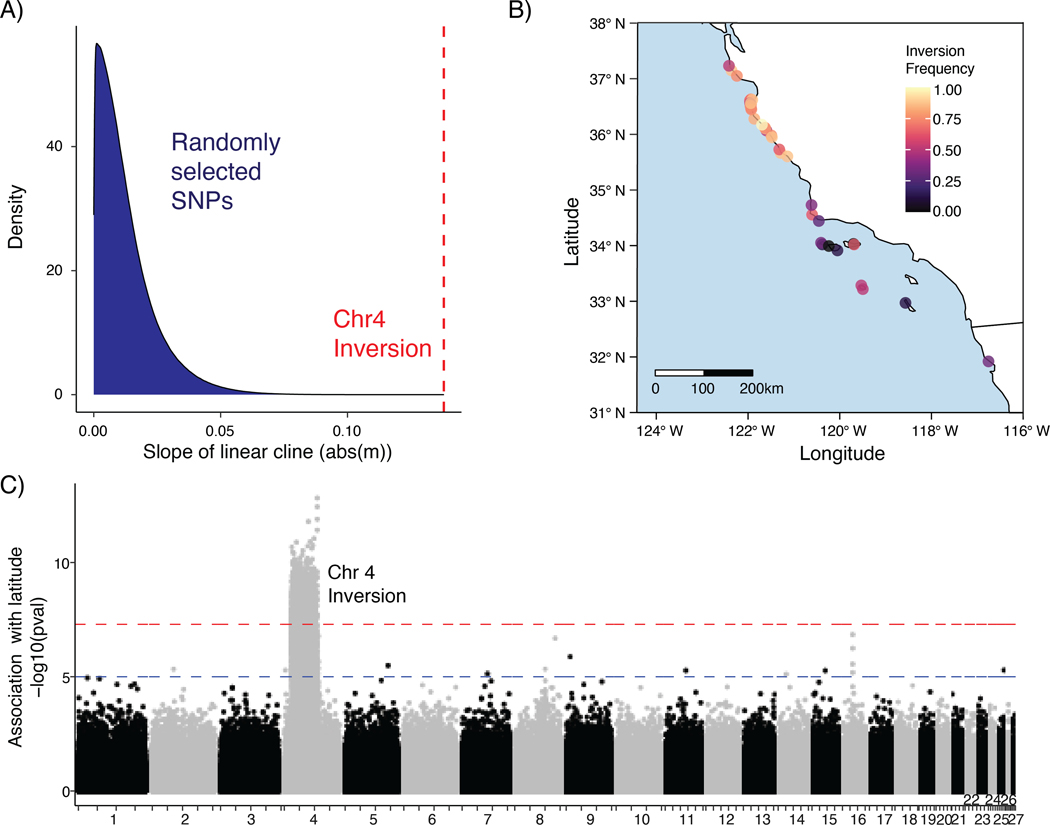
**A)** Distribution of coefficients for linear clines fit to site frequencies for ~14e6 SNPs, as compared to the coefficient for chr4 inversion cline. **B)** Inversion frequency at sample sites visualized across the black abalone range. **C)** GWAS of latitude against genotype for the 27 largest scaffolds in the reference assembly. Blue and red lines correspond to p-values of 1e-05 (suggested association) and 5e-08 (significant association).

We observe a striking north-south cline in the distribution of the chr4 inversion. The linear cline fit to the frequency of the inversion allele is steeper than 100% of linear clines fit to SNPs with minor allele frequency greater than 0.025 and at least 80% of samples genotyped (14.9e6 total; Fig. 3A). Indeed, the frequency of the inversion is significantly associated with latitude, with the inverted allele being more common in the north (Fig. 3 B–C; mean GWAS p-value = 1.59e-08). In contrast, outside of the inversion we do not observe associations with latitude that pass the genome-wide significance threshold, suggesting no or weak spatial structure present in the remainder of the genome (97.4% excluding the inversion).

The inversion cline may be driven by any number of variables that are correlated with latitude (e.g. temperature), making it difficult to pinpoint what, if any, selective forces are underlying this pattern. An analysis of gene content using the genome annotation from the related red abalone (*Haliotis rufescens*), shows 1,030 protein-coding genes contained within the inversion. One gene overlaps with the start coordinates of the inversion and may therefore be disrupted by the rearrangement. However, this gene, identified only as “tetraspanin-9-like”, likely represents a cell surface protein with an unknown function in abalone biology. A gene ontology analysis based on homologues in the Eastern oyster (*Crassostrea virginica*; see Methods) detected enrichment in the inversion for several biological processes, but nothing that indicates a clear association with putative adaptive traits in abalone (Table S1).

### 2.3 Isolation-by-distance (IBD) and isolation-by-environment (IBE)

Excluding the chr4 inversion, we observed that genetic differentiation between collection sites was low, with genome-wide Hudson’s F_ST_ ranging from just 0.02 to 0.07. This limited differentiation across is consistent with the lack of discernible structure in the “no-inversion” PCA (Fig. 2C), suggesting panmixia. Nevertheless, the extent of differentiation between collection sites - however low - may still be correlated with the distance between sites. Therefore, we aimed to determine what physical or environmental factors, if any, may be driving genetic isolation between remaining black abalone populations.

**Figure 4.**
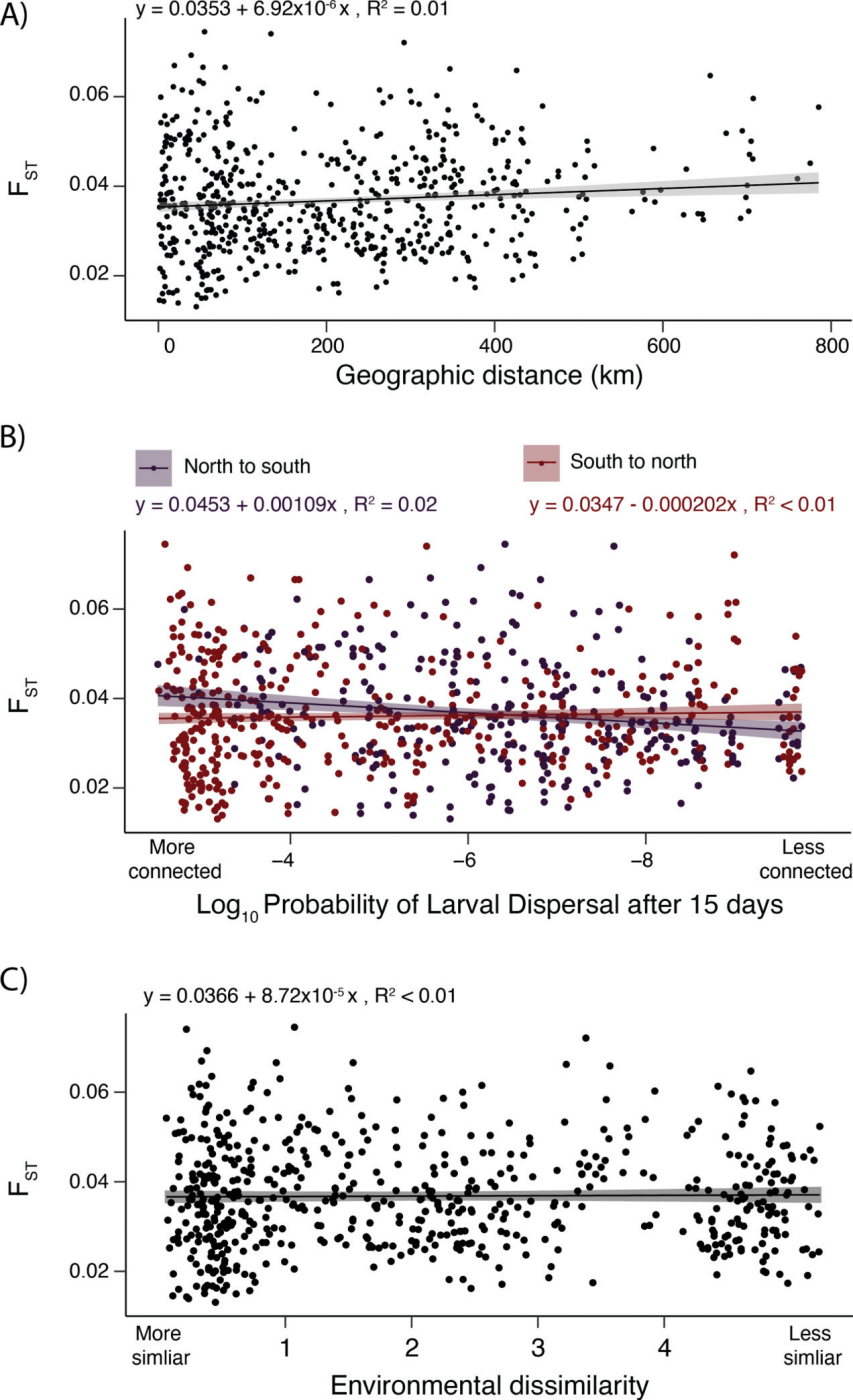
**A)** Isolation-by-Distance: genetic differentiation between sites (F_ST_) as a function of straight-line distance between sites. **B)** Isolation-by-Distance: F_ST_ as a function of the probability of pelagic larvae dispersing (PLD) between sites within 15 days. Results for north-to-south and south-to-north are plotted. **C)** Isolation-by-Environment: F_ST_ as a function of environmental dissimilarity between sites. Environmental dissimilarity encompasses air temperature, sea temperature, and pH.

We observed little relationship between overall genetic differentiation and physical or oceanographic distance between sites, known as isolation-by-distance (IBD; Wright 1943). The relationship between F_ST_ and physical distance was weak but significant, with the linear model showing F_ST_ increasing by only 0.00035 for every 500 km of straight-line distance between sites (R^2^ = 0.01, p < 0.001; Fig 4A). Because black abalone are a marine species and ocean currents on the west coast of North America can be asymmetric, we then modeled the relationship between genetic distance and ‘connectedness’ between sites; that is, the probability of larvae dispersing (PLD) from one site to the next at 5, 10, and 15 days. Even for the 15 day model, which is roughly half the absolute maximum larval duration of black abalone (Morse et al. 1979), we observed almost no relationship between genetic distance and the probability of larval dispersal between sites (north to south: R^2^ = 0.02, p < 0.001; south to north: R^2^ = 0.01, p = 0.279; Fig. 4B). In other words, pairs of sites that are more connected via larval dispersal do not necessarily harbor abalone populations that are more closely related.

We also found no relationship between the extent of genetic differentiation between collection sites and the similarity of their environments, also known as isolation-by-environment (IBE; (Wang and Bradburd 2014). To quantify environmental distance between collection sites, we measured differences in key environmental variables known to affect black abalone fitness. We summarized differences in air temperature, water temperature, and pH between collection sites via principal components analysis (Fig. S2). We found no association between the extent of environmental mismatch between sites - as quantified by environmental PC1 - and genetic distance (R^2^ = 0.01, p = 0.671; Fig. 4C). Therefore, abalone from sites with more similar conditions (e.g. sea surface temperature) are not necessarily more closely related.

### 2.4 Genetic diversity and demography

**Figure 5.**
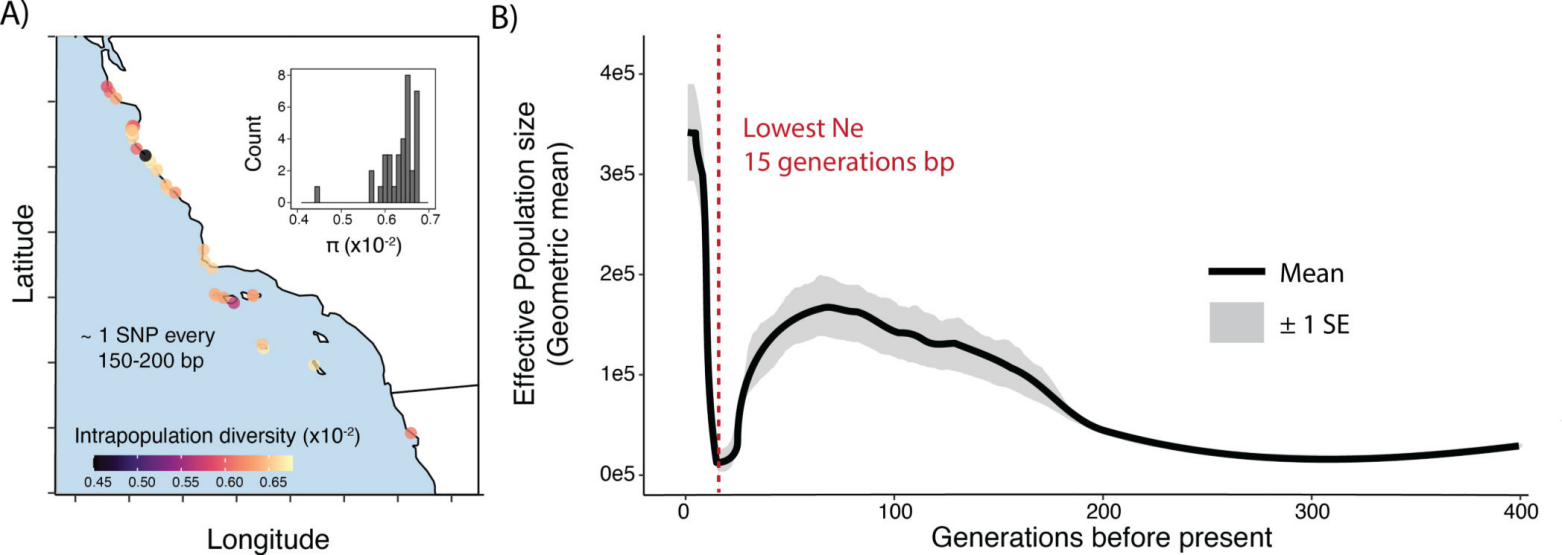
**A)** Intrapopulation genetic diversity (π) visualized by location. Inset corresponds to a histogram of the same values plotted as points on the map. **B)** Effective population size through time as inferred by ‘GONE’ (Santiago et al. 2020), based only on samples with >8X sequencing coverage (n=76). Mean and standard error are shown for original data and 20 jackknife samplings of the genome (subsampling chromosomes, not individuals).

Genetic diversity was uniformly high across our sample sites. We calculated the average number of differences between pairs of genomes at a site (π; (Tajima 1989) at each discrete site in our study (n=35; Fig 1A). Median π is 0.64×10^−2^ (min: 0.45×10^−2^; max: 0.67×10^−2^), equivalent to ~ 1 SNP every 150 bp in the genome. While our per-site sample sizes were small (n=3–4), previous work has shown that small sample sizes may skew estimates of diversity downward (Subramanian 2016), resulting in, if anything, an underestimate of true diversity in our data. Latitude was not significantly associated with π (p=0.47), indicating no clear spatial pattern in the distribution of polymorphism. Even the southern sites most impacted by withering syndrome showed genetic diversity on par with the less impacted northern sites (Fig. 5A).

While the results outlined above highlight surprising genetic diversity in today’s populations, genomic inference of demographic history nevertheless shows an intense bottleneck occurring in the species’ recent past. A first analysis of demography with *SMC*++ (Terhorst, Kamm, and Song 2017), reveals a large effective population size through much of the species’ deeper history (N_e_ > 100,000 individuals ~2e3–1e4 generations before present) that declines to a more moderate size of ~35,000 individuals just 1000 generations ago (Fig. S4). However, as *SMC*++ and related methods have difficulty resolving events in the very recent past (<1000 generations), we also inferred demographic history with *GONE*, an LD-spectrum based approach to infer population size in this time frame (Santiago et al. 2020). Using this approach, we infer an intense demographic bottleneck beginning around 30 generations in the past. Within just 15 generations, effective population size plummets by 90% (mean N_e_ 15–20 gens = 14,680; mean N_e_ 30–35 gens = 113,888; Fig 5B). However, this bottleneck is also brief; after reaching a minimum 15 generations ago, N_e_ is then inferred to recover rapidly, reaching the present day estimate of 3.4e5 individuals.

### 2.5 Prevalence of withering syndrome bacteria and phage hyperparasite

**Figure 6.**
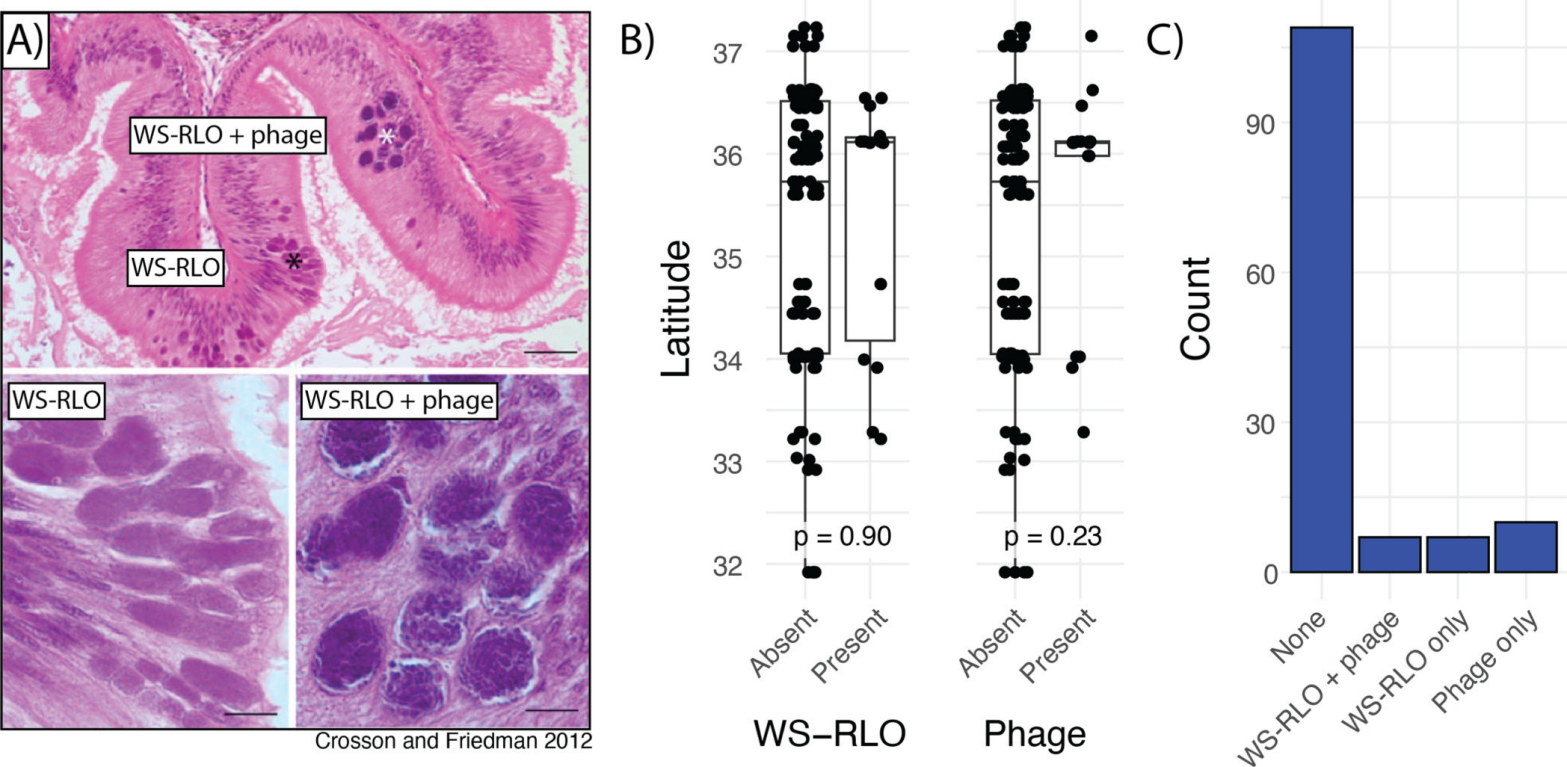
**A)** Representative light micrographs of WS-RLO and phage-infected WS-RLO in the posterior esophagus of black abalone. Figure adapted from (Carolyn S. Friedman and Crosson 2012)**. B)** Association between latitude and presence of WS-RLO or phage. **C)** Observed counts of individuals with for all combinations of WS-RLO and phage presence or absence.

DNA from the bacterial agent of withering syndrome was readily detectable with our swab- based sampling approach, appearing in 10.5% (n = 14) of sequencing libraries. This may be an underestimate, as WS-RLO in its early stages primarily affects the digestive tract, while being readily observable in all tissues later in disease progression (Crosson et al. 2014). However, swab libraries positive for WS-RLO spanned our sampling range (Fig. 6B), suggesting that, while absolute abundance may remain unclear, WS-RLO is nevertheless sporadically present in black abalone across the sampled range. Indeed, there is no significant association between presence or absence of WS-RLO and latitude (Fig. 6B; Welch two-sample t-test p = 0.90). The phage hyperparasite is similarly scarce, and is observed in just 12.8% (n = 17) of sequencing libraries. The presence or absence of phage shows no significant association with latitude either, suggesting that one is just as likely to observe it in northern sites as southern sites (Fig. 6B; Welch two-sample t-test p = 0.23)

Surprisingly, WS-RLO and phage are not always observed together in the same sample (Fig. 6C). 5.3% of black abalone contain both parasites, while 5.3% and 7.5% contain just WS-RLO or phage, respectively. The absence of WS-RLO in phage positive samples is not eliminated after applying more strict or relaxed detection criteria to both parasites.

## 3 Discussion

Despite experiencing a near-extinction level decline in the recent past, black abalone (*Haliotis cracherodii*) harbor high genetic diversity and exhibit almost no population structure. (Fig. 4; Fig. 5). Our estimates of pairwise sequence diversity rank black abalone as more genetically diverse than most vertebrates (Teixeira and Huber 2021; Robinson et al. 2016) and more diverse than organisms with similar biology and life history, including western Pacific abalone (Hirase et al. 2021), which maintain high diversity due to their long-term large effective population sizes. This high diversity is also unstructured across their range (Fig 2C; Fig.4), rejecting previous hypotheses that the negatively buoyant phase and relatively brief larval duration of black abalone would amplify geographic structure (Chambers et al. 2006). Instead, black abalone appear to be panmictic. A similar lack of population genetic structure has been documented in wide-ranging broadcast spawning organisms in California, particularly those with high fecundity, extended spawning periods, and intermediate (2–4 weeks) to long (>8 weeks) planktonic larval stages (Dawson 2001). Therefore, the apparent panmixia of black abalone might be expected given the species’ life history, yet remains surprising given the widespread devastation and local extirpation that withering syndrome caused.

While black abalone were seemingly buffered against a significant loss of genetic diversity during their recent decline, their genomes nonetheless retain a signal of a recent, brief, intense population bottleneck that occurred 15–20 generations ago (Fig. 5B). This signal is intriguing, as the bottleneck’s magnitude - a ~90% decline in N_e_ - is similar in magnitude to the 99% decline attributed to withering syndrome south of Point Conception, and to a lesser extent, the 0–50% decline in populations north of this boundary (Neuman, Tissot, and VanBlaricom 2010). Our model also indicates recovery in recent generations, which agrees with observations of rapid post-WS population growth at some of the California Channel Islands (Kenner and Yee 2022) and expansion in northern sites (Miner et al. 2006). While a recent increase in population size could be reflective of recent gene flow or admixture (Santiago et al. 2020), the lack of population structure outside the inversion, which is not included in this inference, would suggest that this is not a contributing factor. Given these observations, it is tempting to infer that this bottleneck signal directly corresponds to withering syndrome collapse and subsequent recovery. However, the unknown generation time of black abalone makes the translation of generation-based events to real-time events tenuous. Black abalone may reproduce as early as 2–4 years of age - taken strictly as generation time, this would suggest that minimum N_e_ occurred somewhere between 1961 and 1991, a time period roughly corresponding the late 1980s WS collapse (individuals predominantly sampled in 2021; (Neuman, Tissot, and VanBlaricom 2010; Leighton and Boolootian 1963). However, while detailed fecundity data is lacking, black abalone can live as long as 30 years and may reproduce throughout. Depending on the average age of reproduction and the reproductive output of older individuals, generation time could be greater than 2–4 years. Because of this the bottleneck may correspond to an older event, but it should be noted that the most intense fishing pressure in modern times occurred just prior to WS collapse (1970s), revealing no clear candidate for what event may have produced this dramatic signal (Vileisis 2020; Haas et al. 2019; VanBlaricom et al. 2009).

The only signal of strong genetic structure in our data set was driven by a 31MB chromosomal inversion present on chr4 (Fig. 2A–C, Fig 3). The inversion is geographically structured, with the derived (low polymorphism) allele increasing in frequency with latitude (Fig. 3B, Fig. S3). Domination of genome-wide structure by a single locus is rare but has been observed in other taxa with high gene flow and segregating chromosomal rearrangements (Luna et al. 2023; Mérot et al. 2021). These inversion polymorphisms may persist in populations if they are evolving under some form of selection. Inversions can link adaptive alleles, for example, and prevent recombination with maladaptive haplotypes, the latter of which is more likely to occur in high gene flow systems (B. Charlesworth and Barton 2018; Hager et al. 2022; Joron et al. 2011; Kirkpatrick and Barton 2006). The inversions themselves may also be adaptive, for example if an inversion breakpoint disrupts key genes (Küpper et al. 2016; Villoutreix et al. 2021). While in this case we do observe a gene overlapping the first inversion breakpoint at 9.85 Mb, the lack of information regarding this candidate - a tetraspanin-9 like gene - prohibits further investigation at this point.

Although the mechanism by which an inversion influences a phenotype will vary, chromosomal inversions are repeatedly implicated in local adaptation through direct phenotypic associations (Hager et al. 2022; Sanchez-Donoso et al. 2022; Nosil et al. 2023) or associations with environmental variation (Kapun and Flatt 2019; Mérot et al. 2018; Todesco et al. 2022). While no specific phenotype is associated with the black abalone inversion, the significant increase in inversion frequency in northern latitudes (Fig. 3) and the lack of nucleotide diversity in this northern haplotype (Fig. S3) together suggest evolution under natural selection. Given that the northern populations are more resistant to withering syndrome than populations to the south it is tempting to speculate that the inversion is correlated with this resistance. However, the presence of 1,030 genes within the inversion makes gene-based inference of phenotypic effects difficult (see Table S1). Additionally, latitude is strongly correlated with a suite of environmental variables that could be acting as selective forces. Functional data, particularly range-wide transcriptomes and detailed disease phenotypes, will be necessary to determine if the inversion is associated with withering syndrome resistance or an entirely different phenotype.

Although southern black abalone populations have historically been more impacted by withering syndrome, we detected WS-RLO, the agent of withering syndrome, in individuals from both the northern and southern ends of our sampled range (Fig. 6). There was no significant difference in WS-RLO presence according to latitude, indicating that, while perhaps uncommon in healthy black abalone, the bacteria has a wide range that includes northern, colder sites (e.g. Carmel, CA) which have not experienced significant withering syndrome outbreaks (Crosson et al. 2014); Fig. 6B). The phage hyperparasite, while similarly scarce, also showed no significant association with latitude (Fig. 6B). This raises questions as to whether phage presence indeed attenuates WS severity, as has been suggested previously (Carolyn S. Friedman et al. 2014). If true, the phage’s distribution may be expected to align with the historical presence (or absence) of disease, which we do not observe. However, complementary measures of phage and pathogen abundance from the primary tissues affected by the disease (e.g. digestive tract) and more thorough disease phenotypes will be necessary to confirm these observations. Additionally, while the lack of spatial pattern in the presence of both parasites is intriguing, accurate identification of WS-RLO and phage may be constrained by technical barriers, for example inconsistent sequencing effort. Accurately determining the level of endemism of WS-RLO and its association with abiotic (e.g. latitude) and biotic (e.g. phage) factors is key to further informing conservation strategy.

Our results provide guidance for the ongoing management of black abalone along the Pacific coast. The high degree of genetic diversity remaining among populations and the sharing of this diversity - and WS-RLO - across the range indicates that translocation of individuals from healthy populations could be a feasible and low-risk recovery plan. However, these findings also set the stage for future work, in particular research into potential adaptive loci (i.e. chr4 inversion) to better design region-specific management strategies and avoid eroding locally adaptive genotypes. Whether or not adaptation is occurring or incipient growth will continue, these data clearly show that substantial genetic variation persists in today’s populations. This finding alone is an encouraging sign for the species’ recovery prospects.

## 4 Methods

### 4.1 Sample collection

We collected samples in a semi-invasive, non-lethal manner by firmly swabbing the foot of the black abalone with a sterile flocked swab (Puritan, 25-3606-U). Individuals were not removed from the substrate, with the intention that no long-term injuries would be inflicted on the animals. In total, we swabbed 150 abalone between January 24^th^ 2020 and February 27^th^ 2022 at 25 sites between Pebble Beach (37.23, −122.42) and Boat House (34.55, −120.61) California (USA), at nine sites on five of the California Channel Islands (San Clemente, Santa Rosa, San Nicolas, Santa Cruz, and San Miguel; USA) and one site in Ensenada, Mexico (Fig. 1; Table S1). Sites in the United States were sampled under NMFS ESA Section 10 Research Permit 18761, while samples from Ensenada, Mexico were swabbed by a local collaborator in Mexico’s commercial fisheries and transferred as DNA extractions in accordance with USFW and USDA regulations. At the time of swabbing, none of the abalone sampled displayed external signs of WS. We collected swabs in duplicates and stored them in Longmire buffer (100 mM Tris, 100 mM EDTA, 10 mM NaCl, 0.5% SDS, 0.2% sodium azide) to preserve DNA, and then stored them at 4°C. We recorded the size of each individual, as well as whether it was submerged in water at the time of swabbing, its distance to its nearest neighbor, the number of abalone in its sub-site (i.e. crack or crevice containing one or more individuals), and the primary species found at the sub-site.

### 4.2 DNA extraction

We extracted DNA from one of the two duplicate swabs using a modified version of the DNeasy^®^ Blood and Tissue kit (QIAGEN) optimized to recover DNA from swabs. In brief, we incubated the swabs for 2.5-hours at 56°C in 360 μl of Longmire buffer and 40 μl of 20 mg/mL proteinase K. Following the incubation, we transferred the liquid to a fresh tube, spun the swabs at 13,000 rpm in a centrifuge for 1 min, and then transferred any released liquid to the same tube. We increased the volumes of Buffer AL and ethanol to 400 μl, but the rest of the protocol was unchanged. We eluted the DNA in 50 μl of Buffer EB (10 mM Tris), and used 1 μl of extract to quantify DNA concentration with the Qubit dsDNA HS Assay Kit (Invitrogen). We repeated the DNA extraction using the duplicate swab if an extraction’s DNA concentration was too low for quantification. We stored the extractions at −20°C.

### 4.3 Library preparation and sequencing

We prepared the DNA extracts into sequencing libraries following the NEBNext Ultra II FS DNA Library Prep Kit (NEB) according to manufacturers’ instructions but replaced the NEBNext Adapters with Y-Adapters. We incubated the samples for five minutes during the enzymatic fragmentation step, performed a double-sided size selection with a SPRI bead mixture prepared according to (Rohland and Reich 2012) (first clean at 0.26 X and second clean at 0.11 X), and amplified the libraries for 6–8 cycles using dual unique indexes. We eluted each library in 21 μl of 0.1 X TE and quantified the DNA concentration using the Qubit dsDNA HS Assay Kit (Invitrogen) and fragment length on a Fragment Analyzer (Agilent). We then screened each library via low coverage sequencing on an Illumina NextSeq 550 (2 × 150 bp). Libraries were then sequenced on an Illumina Novaseq 6000 (2 × 150 bp) with a target depth of 10–20X genome wide coverage.

### 4.4 Mapping and variant calling

We generated a concatenated reference genome in order to accurately map off-target reads such as those contributed by WS bacteria and phage hyperparasite present in our DNA swabs. This concatenated reference genome included the black abalone reference (Orland et al. 2022) (GenBank accession number GCA_022045235.1), the bacterial *Candidatus Xenohaliotis californiensis* 16S ribosomal RNA gene (also referred to as the withering syndrome rickettsia-like organism or WS-RLO; GenBank accession number AF133090.2) and the WS-RLO phage genome (also referred to as the RLO variant or RLOv; GenBank accession number KY296501.1).

We used the *snpArcher* workflow (Mirchandani et al. 2024), an accelerated workflow for variant calling, to generate high-quality variant calls for downstream analysis. Briefly, sequencing reads were first trimmed of adapter sequence using *fastp* (Chen et al. 2018) and aligned to the concatenated genome using *bwa mem -M -K 10000000* (Heng Li and Durbin 2009). We called individual variants with Sentieon (Kendig et al. 2019) Haplotyper and we performed joint genotyping using Sentieon Genotyper to produce a multisample VCF (variant call format) file. For additional quality control, we used *bcftools* (Danecek et al. 2021) to remove individuals with <2x sequencing depth, sites with minor allele frequency < 0.01, and sites with > 75% missing data. We additionally removed sites that failed a set of standard GATK hard filtering thresholds (Van der Auwera et al. 2013) defined in the *snpArcher* workflow. For SNPs, these filters were *ReadPosRankSum < −8.0, QD < 2.0, FS > 60.0, SOR > 3.0, MQ < 40.0 MQRankSum < −12.5, QUAL < 30.* For INDELs, these filters were *ReadPosRankSum < −20.0, QD < 2.0, FS > 200.0, SOR > 10.0, QUAL < 30*. We additionally removed all indels and retained only biallelic SNPs for downstream analysis, resulting in 66,776,934 SNPs, hereafter referred to as the ‘complete’ SNP dataset. Finally, we masked individual genotypes in this ‘complete’ dataset (i.e. setting ‘./.’) where genotype depth was less than 4 reads.

### 4.5 Population structure analyses

To explore population structure via principal components analysis (PCA), we filtered the complete SNP dataset for sites with minor allele frequency > 0.05 and and less then 10% missing genotypes, then further pruned this set to randomly select SNPs separated by 1kb or more with *bcftools +prune -n 1 -N rand -w 1kb*. This pruning window size was intended to speed up downstream computation, and was also informed by our observation of rapid LD decay (Fig. 2F) which indicated severe reduction in allele frequency correlations in SNPs separated by 1000 bp or more. This filtering resulted in 879,644 total variants. Following this we calculated PCAs at the whole genome level and at genomic regions of interest using the *plink v1.90b7* function *--pca* (Purcell et al. 2007). To further explore the contributions of particular genomic regions to PCA clustering in an unbiased fashion, we conducted a local principal components analysis across the full genome via *lostruct* (Han Li and Ralph 2019). Prior to running this analysis, we refined our original complete SNP dataset to retain only SNPs with minor allele frequency > 0.05 and removed sites with >50% missing data but no window-based pruning in order to capture fine-resolution signals, resulting in 25,036,332 total SNPs. Finally, we ran *lostruct* across the full genome with this dataset, calculating principal component analyses in 5kbp windows.

### 4.6 Chromosomal inversion detection and gene content

Based on the results of our preliminary PCA, we scanned our reference genome assembly - generated from an individual originally from Carmel, CA (Orland et al. 2022) - for potential errors or structural anomalies that might be driving the observed signal. We generated a chromatin-interaction map (contact map) by aligning the Omni-C data to the reference genome (xbHalCrac1.0.p_ctg) with *bwa mem* (Heng Li and Durbin 2009). We processed the alignments using *pairtools* (Open2C et al. 2023), *cooler* (Abdennur and Mirny 2020), and *hicExplorer* (Ramírez et al. 2018) (see Orland et al. 2022 for more details). Finally, we visualized it using *HiGlass* (Kerpedjiev et al. 2018). We inspected the contact map visually and identified two regions in chr4 (scaffold 4) with a high intensity signal off-diagonal that resembled an inversion (Fig. S1). To confirm that this region was an inversion, we searched the PacBio HiFi data generated for the genome assembly for structural variants using *Sniffles* with default parameters (Sedlazeck et al. 2018). We then calculated linkage disequilibrium (LD) for all pairs of SNPs on chr4 using *plink v1.90b7 --r2 inter-chr gz dprime yes-really --ld-window-r2 0* (Purcell et al. 2007).

The black abalone genome currently lacks an annotation. Therefore, to analyze gene content within the inversion, we lifted over the annotation from the red abalone genome (GCF_023055435.1) to the black abalone genome using *liftoff* with default parameters (Shumate and Salzberg 2021). We found that 85.3% of genes lifted over successfully from the red abalone to black abalone genome. In order to perform a Gene Ontology (GO) analysis, we needed to obtain gene identifiers compatible with a mollusk species for which GO analyses could readily be performed, in this case the *Crassostrea virginica* (Eastern oyster) database available on *ShinyGo v0.80* (Ge, Jung, and Yao 2020). To do this, we first used *orthofinder* (Emms and Kelly 2019) with default parameters to identify orthologs between the red abalone genome and the Eastern oyste*r* genome (GCF_002022765.2). We were then able to select Eastern oyster gene identifiers corresponding to genes present in the black abalone chr4 inversion, and performed a GO Biological Process enrichment with this gene set using *ShinyGo.*

### 4.7 Isolation-by-distance (IBD) and Isolation-by-environment (IBE)

To assess relationships between genetic and ecological divergence, we obtained several physical and environmental variables for each site. We calculated physical distance between sites using the function *distm(…,fun = distVincentyEllipsoid)* from the R package *geosphere* (Hijmans 2022). We modeled connectivity between sites using mathematical particles in a Regional Oceanic Modeling System (ROMS). Projections were based on the latitude and longitude where particles were released (i.e. donor site) and arrived (i.e. settlement site) after planktonic larval durations (PLD) of 5, 10, and 15 days.

We collected annual average sea surface temperatures (SST) from loggers at each site from as early as 1999 to 2021. Temperature loggers (HOBO TidbiT and HOBO Pendant from Onset Computer Corporation) were deployed in the low intertidal zone and recorded temperature every 15 minutes. We either downloaded loggers in the field using Onset app (HOBOconnect) or collected and downloaded loggers post-field using Onset software (HOBOware), and exported these data as an ASCII file. We processed the data to separate air temperature and water temperature by comparing the data with tide charts and removing temperatures at times where the tidal height was predicted to be lower than the tidal location of the logger. Daily mean sea water temperature was calculated and then averaged to annual mean sea water temperature.

We also obtained monthly air temperature (AT) values using the function *worldclim_country(“USA”, var=“tavg”, path=tempdir(),res=5)* from the R package *geodata* (Hijmans et al., 2022), selected regions corresponding to our sample locations with *terra::extract(…,method = ‘bilinear’)*, and calculated the mean annual temperature for each site. pH data were averaged annually from monthly collections at a 3 × 3 km resolution from 1990 to 2010 but should reflect the average variation pH among sites (Cheresh and Fiechter 2020). In order to transform SST, AT, and pH per-site values into differences between sites, we generated distance matrices with the base R function *dist(…, method=‘manhattan’)*. Following this, we searched for associations between these aspects of environmental distance using the base R function *prcomp*. After observing strong loadings of all variables on PC1 (Fig S2), we thereafter summarized environmental dissimilarity by a site pair’s PC1 score.

Finally, we calculated genetic distance between sites on the ‘complete’ SNP dataset in 10 kb windows excluding the chr4 inversion. To do this, we used Hudson’s F_ST_ via the *scikit-allel v.1.3.6* function *allel.windowed_hudson_fst()*. To examine relationships between the above mentioned variables and F_ST_, we defined a linear model with the base R function *stats::lm()* (e.g. F_ST_ ~ m*PhysicalDistance + b).

We tested for SNP associations with environmental variables in a GWAS framework using *EMMAX* (Kang et al. 2010). We pruned the complete SNP dataset to one biallelic SNP every 1000 bp in order to speed up runtimes while still sampling frequently along the genome, resulting in 1,026,068 total SNPs. We then generated a kinship matrix from this dataset using *plink2 –make-king square* after masking the chr4 inversion (Chang et al. 2015). Finally, to run *EMMAX*, we supplied the genotype data, the kinship matrix, and the first two genetic principal components from the no-inversion PCA (Fig. 2C) as predictors and supplied a response variable (e.g. latitude) for association testing. Our decision to use the non-inversion PCs as predictors was motivated by our observation that genome-wide PC1 (Fig. 2A) functions essentially as an indication of inversion genotype, representing one non-recombining locus and as opposed to neutral genome-wide structure.

### 4.8 Genetic diversity and demographic inference

We quantified genetic diversity (π) for the set of samples from each site using the full SNP dataset with the function *allel.windowed_diversity(…,size=1000)* from *scikit-allel v.1.3.6*. To then obtain summary statistics comparable to previous studies, we rescaled these values of π by dividing estimates by the sliding window size (1kb). While some monomorphic sites within a given window may in fact represent missing polymorphisms due to issues with sequencing depth or variant calling, this means that these estimates of π are, if anything, going to underestimate true sequence diversity. Finally, we reported per-collection-site values of π (Fig. 5) by calculating the median of the genome-wide set of 1kb windows.

To obtain summaries of linkage disequilibrium we ran *PopLDdecay* (Zhang et al. 2019) on the full SNP dataset with the exception of the chr4 inversion. We then used *GONE*, an LD-spectrum based demographic inference tool, to infer population size through recent time (Santiago et al. 2020). In order to gain sufficient resolution and confidence in our LD spectrum, we only included samples with >8X coverage, resulting in 76 samples in total spread equally across the sampled range. After masking the chr4 inversion and retaining only sites that were variant in the 76 sample ‘demography’ dataset, we converted this input data to the *plink* ped/map format with *plink*. We then ran *GONE* with *script_GONE.sh*, setting default parameters with the exception of *hc=0.10* to evaluate a greater range of recombination rates (e.g. more SNP comparisons) during inference. For lack of fine-scale recombination rate data in this species, we assumed a constant recombination rate of 1 cM/Mb with the parameter *cMMb = 1*, following typical recombination rates found in mollusks (Stapley et al. 2017). We also evaluated the effect of this parameter by setting cMMB = 2, and confirmed a highly similar trajectory but shifted in time towards the present (Fig. S5). Therefore, we settled on the cMMb=1 supported by the literature, and re-ran *GONE* on genomic subsamples consisting of randomly sampled chromosomes, ranging from 50%−80% of the genome.

We also used the same dataset to infer longer term changes in population size with *SMC*++ (Terhorst, Kamm, and Song 2017) to. For this analysis we used the same set of samples and SNPs as with GONE. Because *SMC*++ relies on an accurate identification of heterozygous sites as well as the site frequency spectrum (SFS), we generated conservative ‘mappability’ and ‘depth’ masks for the input data. To generate a mask of low-mappability regions, we used *GenMap* (Pockrandt et al. 2020) on the reference assembly with the parameters ‘*-K 150 -E 4’*, and retained low-quality regions where kmers mapped to three or more places in the genome. We then used a custom script (*bamdepth2bed.py*) to generate depth masks to indicate where more than 30% of samples had 5 or less reads mapping to a position, and therefore where genotype information might be unreliable. We then converted the full SNP vcf files to *SMC*++ format using *vcf2smc*, designated the highest coverage sample of each metapopulation as the ‘Distinguished Individual’ (DI) while masking the inversion, low mappability regions, and low depth regions. Finally we ran *smc++ estimate --timepoints 1e3 1e6 --knots 7 --spline piecewise* to generate demographic histories, providing the human germline mutation rate of 2.5e-8 (Lindsay et al. 2019) for lack of any mollusk germline mutation rate or informative priors. We then generated bootstrap resampled datasets using a custom script (‘*SMC_bootstrap_BW.py*’) and reran the above pipeline for each replicate.

### 4.9 Associations with withering syndrome bacteria and phage hyperparasite

We determined the presence of WS-RLO in our DNA swab libraries using the eDNA community profiling tool *tronko* (Pipes and Nielsen 2022). Specifically, for each library we selected all paired-end reads not mapping to the *H. cracherodii* genome. We then provided the sequence reads and the pre-built 16S DNA reference database (https://zenodo.org/records/7407318) to *tronko* specifying a least common ancestor (LCA) cutoff of 4 (*-c 4*). After examining all hits to taxa in the order *Rickettsiales*, which are obligatory intracellular parasites, we then designated reads mapping to *Candidatus Xenohaliotis californicus 16S* (AF133090.2) and *Haplosporidium sp. endosymbiont AbFoot 16S* (AJ319724.1) as true “positives” for WS-RLO presence in our samples.

To detect the phage hyperparasite of WS-RLO, we capitalized on the availability of a high-quality reference genome (KY296501.1; (Cruz-Flores et al. 2018). We identified reads mapping to the 35,728 bp genome (see 2.4), and designated libraries with >25% of the genome covered by one or more reads as “positive” for the phage, assuming such a profile is unlikely to be generated by spurious mapping from other taxa present in our swabs. This heuristic also allowed us to identify libraries that had disproportionate mapping along the phage genome despite lower sequencing effort, avoiding potential false negatives.

## Supplementary Material

Tab S1

Supinfo
