# Supplementary material for: Limited genomic signatures of population collapse in the critically endangered black abalone (Haliotis cracherodii)
Source: Mol Ecol. 2024 Apr 29;34(23):e17362. doi: 10.1111/mec.17362 (PMC11518883; doi:10.1111/mec.17362)
Supplement: Supplementary file 1 — Supinfo S1. [file MEC-34-e17362-s002.pdf]

Limited genomic signatures of population collapse in the critically endangered black abalone (*Haliotis cracherodii*)

Brock Wooldridge[1,2] † , Chloé Orland[1] † , Erik Enbody[3], Merly Escalona[3], Cade Mirchandani[3], Russell Corbett-Detig[3,4], Joshua D. Kapp[1], Nathaniel Fletcher[1], Karah Cox-Ammann[1], Peter Raimondi[1], Beth Shapiro[1,2,4] *

SUPPLEMENT

Table of contents

Figure S1	Pgs. 2-3
Figure S2	Pg. 4
Figure S3	Pg. 5
Figure S4	Pg. 6
Figure S5	Pg. 7
Table S1	Pg. 8
Caption Extended Data Table 1	Pg. 9

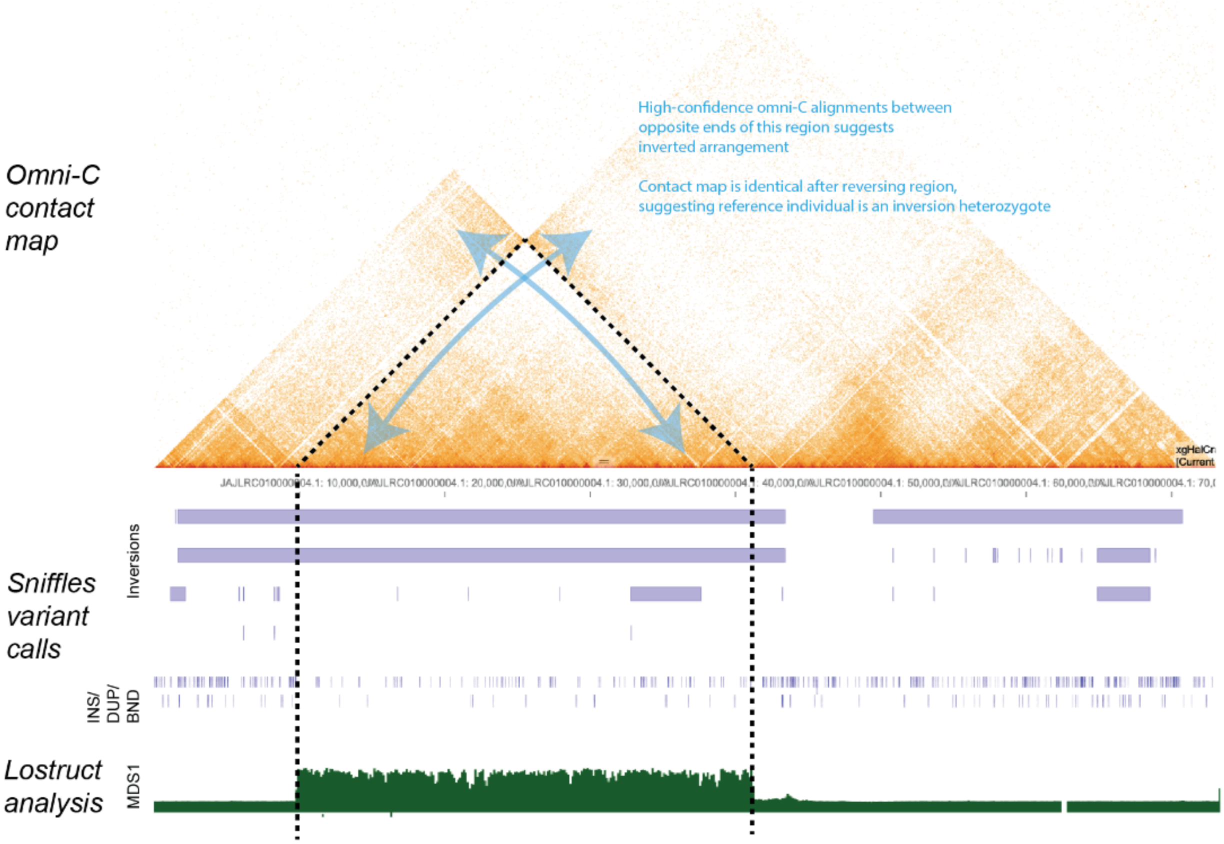

Figure S1. Evidence for a chromosomal inversion in chr4. Top panel displays Omni-C linkage mapping data generated for the reference assembly (Orland et al., 2022) showing an unsolvable region in chr4 that suggests the preference of a chromosomal inversion. Middle panel shows structural variants genotyped based on PacBio long-read variant calls with *Sniffles* (Sedlazeck et al., 2018). INS/DUP/BND refers to insertions, duplications, and translocations, respectively. Proposed inversions overlap imperfectly with boundaries suggested by Omni-C, indicating some uncertainty with inversion coordinates. Bottom panel displays *lostruct* results for chr4 (Li and Ralph, 2019). Each point corresponds to the local genomic structure in 5000 bp genomic

windows, with the “MDS1” position roughly representing deviation in the window from genome-wide structure.

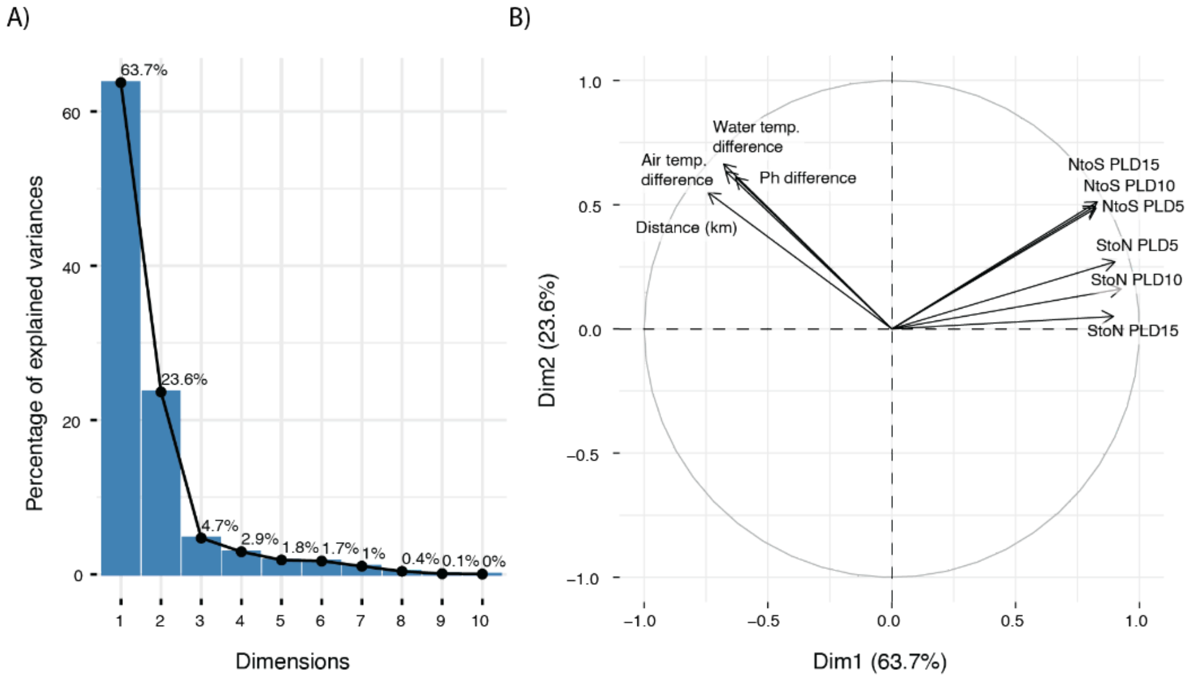

Figure S2. Results of PCA on environmental and physical variables associated with each site. A) Scree plot indicating proportion of variance explained by the first 10 PCs. B) Variable loadings indicating the associations between each variable and the first two PCs.

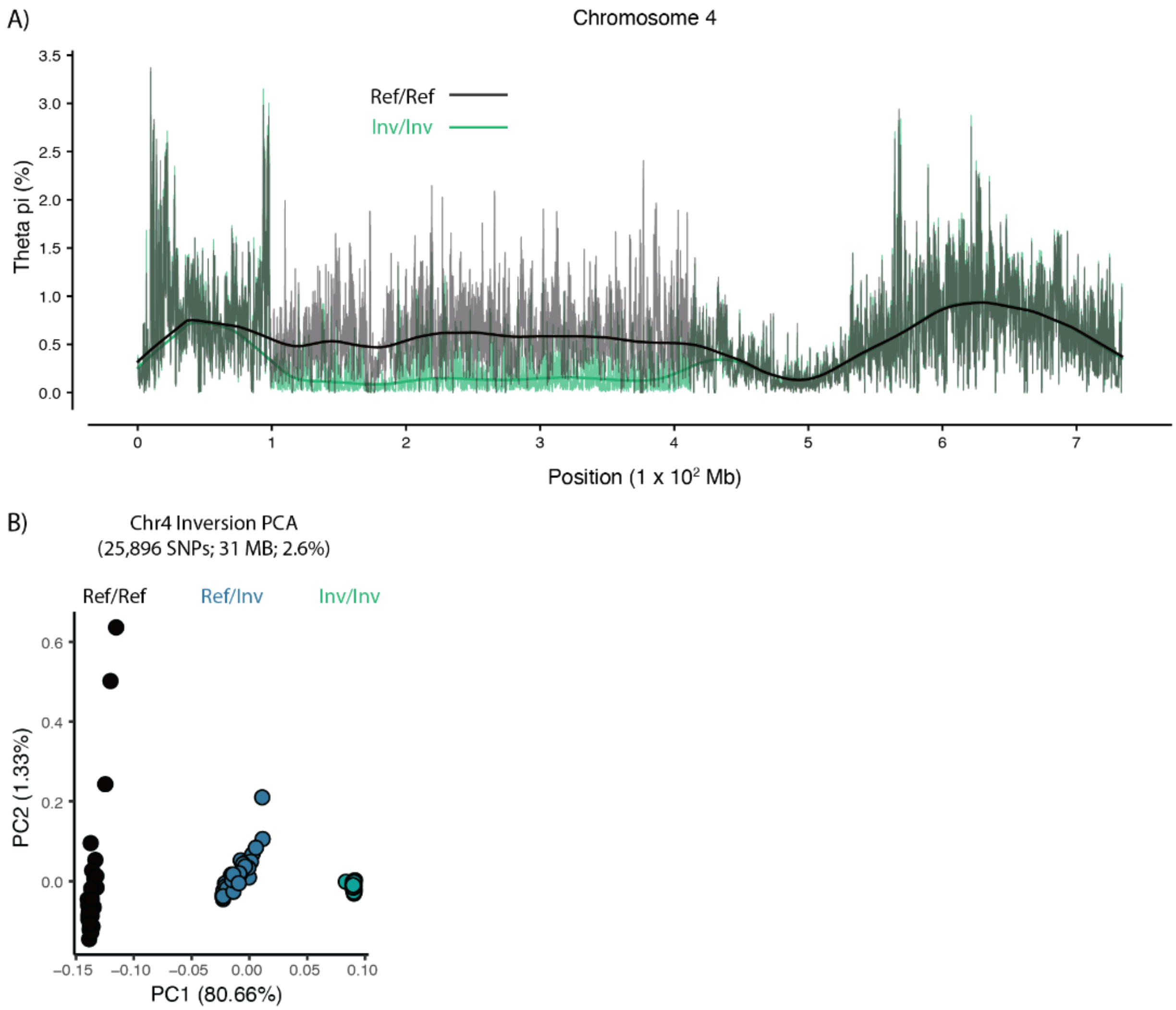

Figure S3. A) Genetic diversity for homozygous reference and homozygous inversion genotypes, where each group is composed of all individuals with that genotype. Diversity (Θ_π) is plotted in 10kb windows, with bold lines representing the loess-smoothed fit. **B)** PCA from Fig. 2B reproduced here to indicate which inversion clusters are being plotted in panel A.

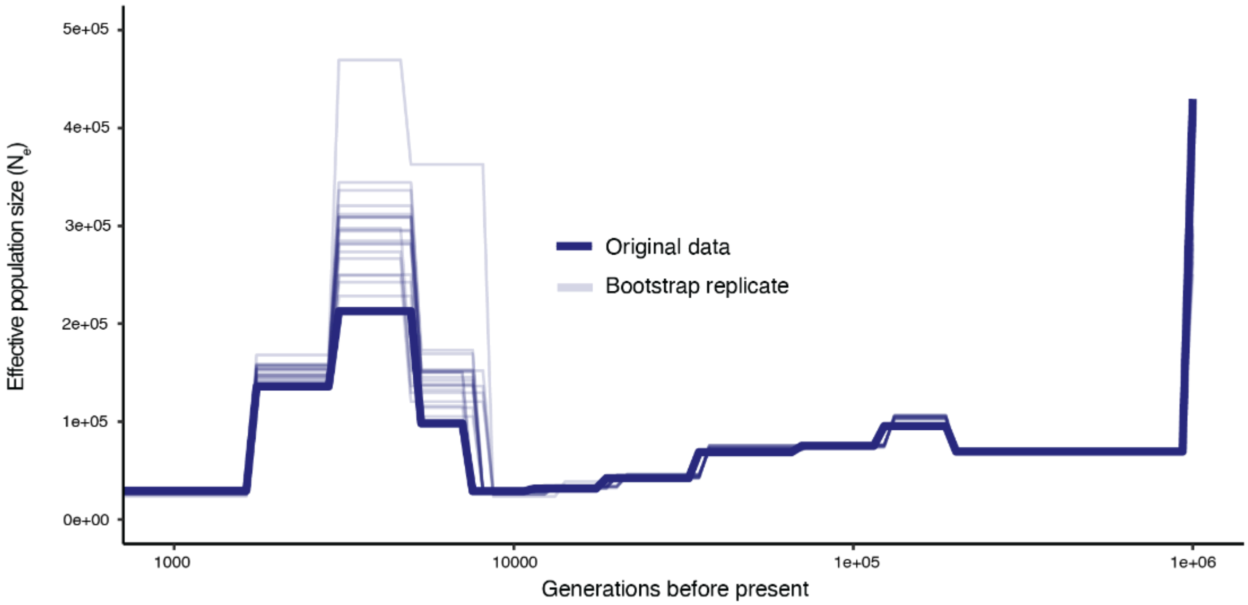

Figure S4. Effective population size through time. Analysis performed with SMC++ (Terhorst et al., 2016) and assuming a germline mutation rate of 2.5e-8. Only samples with >8X sequencing coverage (n=76) are included. Mean and standard error are shown for original data and 20 bootstrap resamplings of the genome (subsampling chromosomes, not individuals).

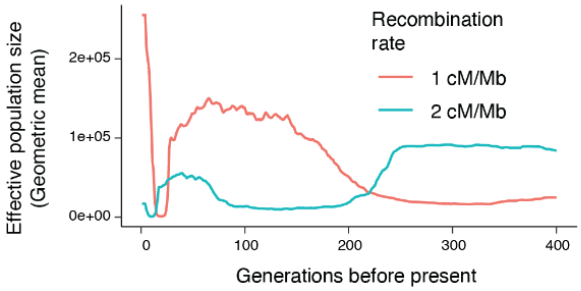

Figure S5. The effect of recombination rate assumptions on recent demographic history as inferred by GONE. Increasing recombination rate from 1 (mollusk-like) to 2 (drosophila-like) parameters shifts events towards recent time and depressed effective population size. See Methods 4.8 for details.

Table S1.

Top GO Biological Processes for genes within the inversion. Analysis based on the *Crassostrea virginica* (Eastern oyster) genome in ShinyGO database, genes identified through homology to genes within black abalone inversion (see Methods). Enrichment FDR = false discovery rate, nGenes = genes in GO category within inversion; Pathway Genes = total number of genes in GO category. Fold Enrichment = percentage of genes in list belonging to a pathway, divided by the corresponding percentage in the background. Pathway = name of GO biological process.

Enrichment FDR	nGenes	Pathway Genes	Fold Enrichment	Pathway
0.00375516	2	3	76.3381643	GO:2001256 Reg. of store-operated calcium entry
0.00375516	2	3	76.3381643	GO:0008612 Peptidyl-lysine modification to peptidyl-hypusine
0.00375516	2	3	76.3381643	GO:0032467 Pos. reg. of cytokinesis
0.00516937	2	4	57.2536232	GO:0006120 Mitochondrial electron transport-NADH to ubiquinone
0.00516937	2	4	57.2536232	GO:0006433 Prolyl-tRNA aminoacylation
0.00516937	2	4	57.2536232	GO:0017183 Peptidyl-diphthamide biosynthetic proc. from peptidyl-histidine
0.00516937	2	4	57.2536232	GO:0031110 Reg. of microtubule polymerization or depolymerization
0.00976874	2	6	38.1690821	GO:0045292 MRNA cis splicing-via spliceosome
0.00976874	2	6	38.1690821	GO:0032088 Neg. reg. of NF-kappaB transcription factor activity
0.01168306	2	7	32.7163561	GO:0006269 DNA replication-synthesis of RNA primer
0.00297969	3	11	31.229249	GO:0008380 RNA splicing
1.72E-05	8	66	13.8796662	GO:0000398 MRNA splicing-via spliceosome
0.01168306	3	26	13.2123746	GO:0090501 RNA phosphodiester bond hydrolysis
0.00168585	5	45	12.7230274	GO:0006351 Transcription-DNA-templated
0.00297969	5	55	10.4097497	GO:0018108 Peptidyl-tyrosine phosphorylation
0.01168306	4	57	8.03559624	GO:0006338 Chromatin remodeling
0.0062702	6	117	5.87216648	GO:0006886 Intracellular protein transport
0.0001289	16	448	4.08954451	GO:0006468 Protein phosphorylation
0.00012377	19	597	3.64428422	GO:0016310 Phosphorylation
0.00875816	13	530	2.80866831	GO:0007165 Signal transduction

Extended Data Table S1. List of swabbed samples and collection locations. Alternate name = more informative shorthand name, where applicable; Site = collection site shorthand; Longitude, Latitude = coordinates with ~1km accuracy; QCpassing = whether or not sample generated sufficient endogenous DNA for genomic analyses.
